# Multiple Chronic Conditions, Delayed Medical Care and Hospitalization: A Comparison Between the United States and Taiwan

**DOI:** 10.34172/ijhpm.9164

**Published:** 2026-02-17

**Authors:** Chen-Yang Wang, Ching-Ching Claire Lin, Raymond N. Kuo, Joshua M. Liao

**Affiliations:** ^1^Institute of Health Policy and Management, College of Public Health, National Taiwan University, Taipei, Taiwan.; ^2^Population Health Research Center, National Taiwan University, Taipei, Taiwan.; ^3^Master of Public Health Degree Program, College of Public Health, National Taiwan University, Taipei, Taiwan.; ^4^Department of Internal Medicine, UT Southwestern Medical Center, Dallas, TX, USA.

**Keywords:** Multiple Chronic Conditions, Delayed Medical Care, Hospitalization, Access to Care, Taiwan, United States

## Abstract

**Background:**

Delays in medical care can be especially critical for individuals with multiple chronic conditions (MCCs). The United States and Taiwan, with vastly different healthcare systems, offer contrasting contexts for access to care. This study aims to examine the relationship between MCCs, delayed medical care and hospitalization in the US and Taiwan.

**Methods:**

This analysis used data from the US National Health Interview Survey (NHIS) 2021 (n=29 482) and the Taiwan Social Change Survey (TSCS) 2021 health module (n=1604). We estimated multivariable logit regression models and calculated differential effects of MCCs status (no chronic conditions, one chronic condition, MCCs) on outcomes. Precision measures were estimated with delta method. All analyses for the US population incorporated applicable complex survey design and weighting, and for the Taiwan population incorporated weighting when appropriate.

**Results:**

Compared to those with no chronic conditions, individuals in the US with one chronic condition (2.0 percentage-points, *P*<.001) or MCCs (3.6 percentage-points, *P*<.001) had a higher likelihood of delayed care due to costs. In Taiwan, delayed care was less likely among individuals with one chronic condition (5.6 percentage-points, *P*=.08) or MCCs (9.5 percentage-points, *P*=.02), compared to individuals with no chronic conditions. Furthermore, individuals with MCCs or one chronic condition are associated with higher hospitalization in both the US (6.1 percentage-point, *P*<.001; 1.6 percentage-point, *P*=.001, respectively) and Taiwan (15.7 percentage-point, *P*<.001, 3.8 percentage-point, *P*=.08, respectively), although the differential effect of one chronic condition in Taiwan did not reach statistical significance.

**Conclusion:**

Analyzing data from two national health systems, this analysis shows differing relationships between MCC status and delayed care, suggesting a possible bidirectional effect. As both regions undergo reforms—US efforts to improve coordination and Taiwan’s rising risk of fragmented care—these findings offer insights relevant to policy-makers and health system leaders beyond each country’s context.

## Background

**Key Messages**

**Implications for policy makers**

- Health policy can mitigate the challenge of delays in medical care among individuals with chronic conditions.
- In systems with high accessibility and minimal cost barriers such as Taiwan, individuals with one chronic condition may be less likely to delay care than individuals with no chronic conditions.
- In systems with more variable or significant care barriers such as the US, individuals with chronic conditions may be more likely to delay care due to cost.

**Implications for the public**

People with chronic conditions often need more medical care but may face greater challenges receiving such care. Comparing patients from the United States and Taiwan, this study found that while patients with chronic conditions were more likely to delay care due to costs than patients without any chronic conditions in the US, patients with chronic conditions were less likely to do so than patients without any such conditions in Taiwan. These findings highlight the key role of affordability, and the potential in addressing cost barriers, in determining if and how patients with chronic conditions access care.

Delays in care is a vital universal indicator of healthcare access – the ability for a patient to see a qualified health provider within a reasonable period of time.^1^ Across healthcare systems, patients can encounter obstacles accessing healthcare due to multiple factors,^2^ such as financial constraints, distance to healthcare facilities, as well as physical or mental health challenges, among others, with negative impacts on health outcomes and healthcare costs for patients and the healthcare system.^3-5^

 In both the US and Taiwan, delays in care are a particularly critical issue for individuals with multiple chronic conditions (MCCs). A high priority group, this population has grown rapidly in the past few decades, particularly among the elderly.^6,7^ Over half of US adults have at least one chronic condition, and more than a quarter have MCCs.^8^ In Taiwan, nearly 40% of individuals aged 20 to 64 have at least one chronic condition, with 17% classified as having MCCs.^9^ Among the Taiwan elderly population, the prevalence is even higher, with over 80% living with at least one chronic condition and more than 60% affected by MCCs.^10^ Individuals with MCCs represent a high-need population that incurs significant healthcare costs while potentially receiving lower quality of care at the same time.^11-13^ In both the US and Taiwan, health needs and risk for care fragmentation could lead to unplanned acute care, such as hospitalization. In turn, ensuring healthcare access is a critical policy and practice priority.

 Though the US and Taiwan possess very different healthcare systems, the two countries share challenges caring for individuals with MCCs. In the US, this population comprises 67% of all spending for national payers such as Medicare.^14^ The negative impacts of care fragmentation are the greatest among individuals with MCCs given their multiple care needs. In Taiwan, patients see physicians 14.3 times a year on average,^15^ with high utilization disproportionately attributable to individuals with MCCs. Without a strong primary care referral system or managed care functions, individuals with MCCs can also potentially experience care fragmentation while representing the largest resource utilizers in Taiwan’s healthcare system.^16^

 Given these issues, there is a critical need to assess the relationship between MCCs and delays in care. Individuals with MCCs typically have greater medical needs than the general population. However, the relationship between chronic condition status and healthcare-seeking behaviors remains unknown, particularly when comparing countries with vastly different healthcare systems.

 Two opposing theoretical frameworks describe this potential relationship. “The competing demands hypothesis” suggests that the management of multiple conditions competes for limited resources—such as time, attention, and financial resources—which may lead to delayed care for other health needs.^17^ Conversely, “the surveillance hypothesis” argues that patients with MCCs have more frequent contact with the healthcare system, which lowers the threshold for seeking care and reduces delays.^17^ This study hypothesized the financial burden in the US system may make the “competing demand”’ effect more dominant compared to the Taiwan system, and the circumstances of Taiwan might be explained by “surveillance hypothesis.”

 The objective of this study was to fill existing knowledge gaps and investigate the effect of MCCs in the US and Taiwan. Particularly, this study compared the effect of MCCs on access and resource utilization by analyzing two specific outcomes of interest, delay in care and hospitalization.

## Methods

###  Sampling and Data Source

 Our analysis used 2021 data from two surveys: the US National Health Interview Survey (NHIS) 2021^18^ and the Taiwan Social Change Survey (TSCS) 2021 health module.^19^ Our sample included all survey respondents aged 18 or older. In the NHIS, respondent answers to delays in care as “refused,” “not ascertained” or “don’t know” were considered missing data, and would not be used in regression models. In the TSCS, respondent answers to delays in care as “not ill or injured during the last 12 months” would not be used in analysis. Hospitalization was also dichotomized into as yes versus no. Responses such as “Refused,” “Not ascertained,” and “Don’t know” or “Skip” (due to following questions in TSCS) were considered as missing data. Supplementary file 1 presented the definition of key variables and the corresponding survey questions from NHIS and TSCS, and Supplementary file 2 presented the sample selection process.

###  Exposure of interest

 The exposure of interest in this study was MCC status: no chronic conditions, one chronic condition, or MCCs (0, 1, and ≥2 conditions). In NHIS, respondents were considered with MCCs if they responded yes to any of the following conditions: hypertension, arthritis, cancer, diabetes, coronary heart disease, angina, myocardial infarction, emphysema or chronic obstructive pulmonary disease, stroke, kidney disease, hepatitis, and psychologic distress.^20,21^ Psychologic distress was measured by the Kessler-6 score (range, 0-24), with a sum score of 13 to 24 defined as being psychologically distressed.^20,21^ In TSCS, respondents were considered with MCCs if they respond yes to one of the following conditions: hypertension, arthritis, cancer, diabetes, heart disease, chronic obstructive pulmonary disease, stroke, kidney disease, hepatitis, and mental illness. This study employed a simple count to define MCC status. This operationalization is a standard approach in survey-based health service research and has been widely accepted and utilized in prior survey studies.^22,23^

###  Outcome Variables

 This analysis included two outcomes of interest: self-reported delays in medical care and hospitalization. For the US sample, we analyzed responses to NHIS questions about whether respondents “delayed getting medical care because of the cost in the past 12 months” and had “ever been hospitalized overnight in the past 12 months.” Responses were dichotomized (yes/no) for both delays in care and hospitalization.

 For the Taiwan sample, we analyzed responses to TSCS questions about whether individuals had “ever refrained from going to see a doctor in the previous 12 months” and “ever been in a hospital or a clinic as an inpatient overnight in the previous 12 months.” Responses were also dichotomized (yes/no) for both delays in care and hospitalization. We acknowledged that the two questions for delay in care from US and Taiwan data are not exactly identical, but financial barriers are a primary reason for delayed care in the US, and previous studies using the same survey have defined unmet medical needs and healthcare accessibility based on the same question employed in this study.^24,25^ Given this context, we consider this question to serve as a broader measure of delays in care in the US. In addition, since Taiwan’s National Health Insurance provides extensive services, financial burden is generally not a primary barrier to care. Instead, hidden costs—such as travel time and limited sick leave days—may contribute to delays in seeking medical care in Taiwan. Therefore, the Taiwan survey question on delayed care was also framed more broadly to capture these factors.^19^

###  Covariates

 We included similar covariates in both US and Taiwan analyses, selected based on previous studies exploring their relationship and heath care accessibility.^26,27^ In NHIS, the variables were age (18-64 or 65+), gender (female or male), general health status (excellent, very good, good and fair/poor), rurality of residence (urban or rural), region (northeast, midwest, south and west), educational attainment (less than a high school education, general educational development/high school, college, Bachelor’s degree and graduated), race and ethnicity (Hispanic, non-Hispanic white, non-Hispanic black and other races), marital status (currently married or not), employment status (employed and unemployed), insurance type (public insurance only, private insurance only, having both public and private insurance and non-public non-private insurance), having a usual place to go for care (yes, no, or more than one), and household income status (ratio of family income to poverty threshold, categorized as <1.49, 1.50-2.49, 2.50~4.00 or >4.00). Analogous variables were assessed from the TSCS, with adjustments as necessary (eg, race/ethnicity and usual source of care information not available in TSCS; region defined as Taipei, north, central, south, Kao-Ping, and east; no respondent has private insurance only in Taiwan; household income status was divided into four groups to facilitate cross-national comparisons: first group (<US $16 000), second group (US $16 000–32 000), third group (US $32 000–72 000), fourth group (>US $72 000)).

###  Data Analysis

 We calculated weighted socio-demographic characteristics and described MCC status in both the US and Taiwan samples. Among individuals with one or more chronic condition, we also estimated the frequency of disease combinations. All descriptive estimations adopted appropriate weighting to reflect the original sampling frame.

 We estimated multivariable logistic regression models, adjusted for covariates noted above, for association between MCC status and the two study outcomes. The marginal effect of MCC status and other covariates on study outcomes were estimated as percentage-point change, with standard errors estimated using the delta method. All analyzes were performed using Stata version 17 (StataCorp LLC, College Station, TX, USA).

## Results

###  Sample Characteristics and Prevalence of Chronic Conditions in the US and Taiwan

 Our samples included 29 482 and 1604 individuals for the US and Taiwan samples, respectively (Table 1). The weighted percentage of the elderly in the samples from the US and Taiwan were similar (22.1% in the US, 20.9% in Taiwan), and slightly more than half of the total population was female (51.7% in the US, 51.4% in Taiwan). Taiwanese respondents had lower educational attainment (33.7% Bachelor’s in the US vs. 28.9% Bachelor’s in Taiwan; 13.1% Graduate in the US vs. 7.9% Graduate in Taiwan; 9.5% Under grade 12 in the US vs. 24.9% Under grade 12 in Taiwan), and lower percentage of family with higher income—43.1% of respondents with family income: poverty ratio of >4 which was around $70 000 in the US compared to only 6.4% of respondents with family income above $72 000 in Taiwan (according to US Department of Health and Human Services, the poverty threshold annually for a household of two persons was $17 420 in 2021^28^). In addition, Taiwanese respondents were more likely to self-report fair/poor health status (13.6% in the US vs. 42.5% in Taiwan).

**Table 1:** Characteristics of Analytical Sample in the US and in Taiwan, 2021

**Characteristics**	**United States**		**Characteristics**	**Taiwan**	
	**No. in Sample Total** **(Unweighted) (n = 29 482)**	**Weighted %**		**No. in Sample Total (Unweighted)** **(n = 1604)**	**Weighted %**
Age, years			Age, years		
18-64	20 519	77.6	18-64	1244	78.8
65+	8877	22.1	65+	356	20.9
Refused/Don’t know	99	0.3	Refused/Don’t know	4	0.3
Gender			Gender		
Female	16 102	51.7	Female	849	51.4
Male	13 378	48.3	Male	755	48.6
Refused/Don’t know	2	0.0			
General health status			General health status		
Excellent	6657	24.6	Excellent	52	3.2
Very good	10 105	34.0	Very good	394	25.3
Good	8350	27.8	Good	474	29.0
Fair/Poor	4357	13.6	Fair/Poor	684	42.5
Refused/Don’t know	13	0.0			
Geographic classification			Geographic classification		
Urban	25 209	86.7	Urban	1258	79.4
Rural	4273	13.3	Rural	336	20.0
			Refused/Don’t know	10	0.6
Region			Region		
Northeast	4775	17.5	Taipei	441	29.1
Midwest	6327	20.8	North	225	16.8
South	10 731	37.9	Central	321	19.1
West	7649	23.8	South	237	13.6
			Kao-Ping	269	15.6
			East	111	5.8
Education			Education		
Under grade 12	2533	9.4	Under grade 12	404	24.9
GED/High school	7251	28.2	GED/High school	448	29.2
College	4453	15.0	College	166	9.1
Bachelor's degree	10 660	33.7	Bachelor's degree	456	28.9
Graduate	4433	13.1	Graduate	130	7.9
Refused/Don’t know	152	0.6			
Race and ethnicity					
Non-Hispanic White	19 658	62.8			
Hispanic	4081	16.9			
Non-Hispanic Black/African	3160	11.7			
Others	2538	8.6			
Marital status			Marital status		
Married/partner	15 263	58.4	Married/partner	827	52.3
Unmarried	14 219	41.6	Unmarried	727	47.7
Employment status			Employment status		
Employed	16 461	60.1	Employed	1065	67.2
Unemployed	12 043	36.5	Unemployed	539	32.8
Refused/Don’t know	978	3.4			
Insurance type			Insurance type		
Public insurance only	4303	11.1	Public insurance only	512	32.8
Private insurance only	14 424	53.5	Public + private	1081	66.3
Public + private	7586	22.7	Non-public, non-private	11	0.9
Non-public, non-private	3169	12.7			
Household income, percent of federal poverty level			Household income, category by quartile		
<150%	5552	18.9	1st group (under US $16 000 annually)	417	15.5
150% to <250%	4889	16.7	2nd group (US $16 000-32 000)	386	23.9
250% to <400%	6109	21.3	3rd group (US $32 000-72 000)	396	25.1
400% or more	12 932	43.1	4th group (over US $72 000)	103	6.4
			Refused/Don’t know	302	19.2
Have a usual place to go for care					
No	2601	10.3			
Yes	26 168	87.2			
More than 1	496	1.8			
Refused/Don’t know	217	0.7			

Abbreviation: GED, general education degree.

 MCCs were more prevalent in the US than in Taiwan (28.9% of individuals in the US vs. 12.6% of individuals in Taiwan) (Table 2). The percentage of individuals with one chronic condition in the US was also slightly higher (25.9% in the US vs. 23.7% in Taiwan). The three most prevalent conditions in the US were hypertension (57.3%), arthritis (38.8%) and cancer (18.0%). On the other hand, in Taiwan, the three most prevalent conditions were hypertension (49.5%), diabetes (24.7%) and heart disease (14.3%). Disease combinations also differed: within two chronic conditions sample, the three most common combinations of two chronic conditions in the US were hypertension and arthritis (26.2%), hypertension and diabetes (12.3%), and hypertension and cancer (9.1%); the three most common combinations in Taiwan were hypertension and diabetes (28.2%), hypertension and heart disease (11.1%) and hypertension and arthritis (4.2%) (See Supplementary file 3).

**Table 2:** The Combination of Diseases in the United States and in Taiwan

**The Frequency of MCC Status**					
**United States (N = 29 482)**			**Taiwan (N = 1604)**		
**MCC Status**	**N**	**Weighted %**	**MCC Status**	**N**	**Weighted %**
MCC	11 741	28.9%	MCC	210	12.6%
Only with one CC	7628	25.9%	Only with one CC	398	23.7%
Without any CC	10 113	45.2%	Without any CC	996	63.7%

Abbreviations: MCC, multiple chronic condition; CC, chronic condition.

###  Differential Effects of Chronic Conditions on Delayed Care in the US and Taiwan

Table 3 illustrates the differential effect of MCC status on outcomes in two countries. Compared to those without any chronic conditions, individuals with one condition or MCCs were more likely to report delayed care due to cost in the US (2.0 percentage point, *P* < .001; 3.6 percentage point, *P* < .001, respectively), but were less likely to report delayed care in Taiwan (5.6 percentage point, *P* = .08; 9.5 percentage point, *P* = .02, respectively). Compared to those who reported excellent health status, individuals with very good, good and fair/poor in the US were more likely to report delayed care due to cost (1.4 percentage point, *P* = .001; 3.6 percentage point, *P* < .001; 8.4 percentage point, *P* < .001, respectively) in the US. While the pattern of effect was similar in Taiwan, the effects of different health status on delayed care were not significant.

**Table 3:** Weighted Marginal Effect Estimates of Multiple Chronic Condition on Delayed Medical Care and HOSPITALIZATION

**United States**							**Taiwan**						
**Outcome Variables**	**Delayed Care Due to Cost in the Past 12 months (n = 29 251)**			**Hospitalized overnight in the Past 12 months (n = 29 229)**			**Outcome Variables**	**Refrained From Going to a Doctor in the Past 12 months (n = 1482)**			**Hospitalized Overnight in the Past 12 months (n = 1195)**		
	**ME**	**SE**	***P*** ** Value**	**ME**	**SE**	***P*** ** Value**		**ME**	**SE**	***P*** ** Value**	**ME**	**SE**	***P*** ** Value**
MCC status							MCC status						
No CC	[Reference]			[Reference]			No CC	[Reference]			[Reference]		
With only one CC	0.020	0.005	<.001	0.016	0.005	0.001	With only one CC	-0.056	0.032	.08	0.038	0.022	.08
MCC	0.036	0.005	<.001	0.061	0.006	<.001	MCC	-0.095	0.042	0.02	0.157	0.038	<.001
Age (65+)	-0.055	0.004	<.001	-0.011	0.005	.045	Age (65+)	-0.136	0.036	<.001	-0.018	0.024	.46
Male	-0.022	0.003	<.001	-0.021	0.004	<.001	Male	-0.001	0.026	.96	-0.033	0.019	.08
General health status							General health status						
Excellent	[Reference]			[Reference]			Excellent	[Reference]			[Reference]		
Very good	0.014	0.004	.001	0.013	0.004	.004	Very good	0.013	0.080	.87	(Not estimable)		
Good	0.036	0.005	<.001	0.033	0.005	<.001	Good	0.092	0.080	.25	(Not estimable)		
Fair/Poor	0.084	0.008	<.001	0.093	0.008	<.001	Fair/Poor	0.099	0.079	.21	(Not estimable)		
Urban	-0.009	0.004	.059	-0.004	0.005	.43	Urban	-0.079	0.036	.03	0.029	0.022	.19
Region							Region						
Northeast	[Reference]			[Reference]			Taipei	[Reference]			[Reference]		
Midwest	0.018	0.006	.008	0.009	0.006	.11	North	0.035	0.043	.42	0.010	0.030	.73
South	0.012	0.006	.053	0.006	0.005	.23	Central	0.014	0.039	.72	0.026	0.028	.36
West	0.020	0.007	.003	-0.004	0.005	.48	South	-0.046	0.042	.27	0.003	0.030	.93
							Kao-Ping	-0.061	0.039	.12	0.020	0.028	.47
							East	-0.069	0.054	.20	0.025	0.039	.52
Education							Education						
Under 12 grades	[Reference]			[Reference]			Under 12 grades	[Reference]			[Reference]		
GED/High school	0.008	0.006	.14	0.015	0.006	.02	GED/High school	0.039	0.038	.31	0.007	0.025	.79
College	0.022	0.007	.001	0.006	0.007	.40	College	0.159	0.053	.003	-0.034	0.031	.27
Bachelor's degree	0.026	0.06	<.001	0.012	0.007	.09	Bachelor's degree	0.031	0.042	.46	0.015	0.031	.62
Graduate	0.017	0.008	.03	0.020	0.008	.01	Graduate	0.096	0.059	.10	0.045	0.049	.35
Race and ethnicity													
Non-Hispanic White	[Reference]			[Reference]									
Hispanic	-0.017	0.005	.002	0.003	0.006	.65							
Non-Hispanic Black	-0.020	0.005	<.001	0.010	0.006	.12							
Others	-0.022	0.006	<.001	-0.010	0.007	.17							
Marital status	-0.002	0.004	.68	0.016	0.004	<.001	Marital status	0.032	0.027	.25	0.043	0.020	.03
Employment status	-0.000	0.004	.93	-0.016	0.004	<.001	Employment status	0.037	0.032	.24	-0.033	0.023	.15
Insurance type							Insurance type						
Public only	[Reference]			[Reference]			Public only	[Reference]			[Reference]		
Private only	0.009	0.009	.30	-0.020	0.008	.01	Public + private	-0.033	0.031	0.29	-0.013	-0.022	0.54
Public + private	-0.027	0.007	<.001	0.009	0.006	.10	Non-public non-private	0.111	0.175	.53	(Not estimable)		
Non-public non-private	0.087	0.012	<.001	-0.024	0.009	.01							
Household income							Household income						
<150%	[Reference]			[Reference]			1st group	[Reference]			[Reference]		
150% to <250%	-0.009	0.008	.25	-0.008	0.006	.24	2nd group	0.022	0.039	.58	-0.025	0.029	.38
250% to <400%	-0.026	0.007	<.001	-0.008	0.006	.19	3rd group	0.045	0.042	.29	-0.017	0.032	.59
400% or more	-0.052	0.007	<.001	-0.008	0.006	.20	4th group	0.070	0.063	.27	-0.057	0.041	.17
Have a usual place to go for care													
No	[Reference]			[Reference]									
Yes	-0.048	0.008	<.001	0.017	0.007	.02							
More than 1	-0.023	0.06	.15	0.030	0.015	.043							

Abbreviations: ME, marginal effects; SE, standard error; MCC, multiple chronic condition; GED, general education degree; CC, chronic condition.

 Hispanic ethnicity was associated with lower adjusted likelihood of delayed care due to cost (1.7 percentage point; *P* = .002) as compared with non-Hispanic White reference group. Compared to those with public insurance only, those who had both public and private insurance were associated with lower adjusted likelihood of delayed care due to cost in the US (2.7 percentage point, *P* < .001); however, those without any type of insurance were had higher adjusted likelihood of delayed care (8.7 percentage point, *P* < .001). In Taiwan, insurance types were not associated with delayed care. Compared to those without a usual place for care, having preference place for care was associated with lower adjusted likelihood of delayed care due to cost in the US.

###  Association Between Chronic Conditions and Hospitalization in the US and Taiwan

 MCC status was also associated with hospitalization. In particular, compared with those without any conditions, individuals with one chronic condition or MCCs were more likely to experience hospitalization in both the US (1.6 percentage point, *P* = .001; 6.1 percentage point, *P* < .001, respectively) and Taiwan (3.8 percentage point, *P* = .078; 15.7 percentage point, *P* < .001, respectively). Compared to those who reported excellent health status, individuals with very good, good and fair/poor in the US were more likely to experience hospitalization (1.3 percentage point, *P* = .004; 3.3 percentage point, *P* < .001; 9.3 percentage point, *P* < .001, respectively). Lastly, compared to those having public insurance only, individuals who used private insurance only, or individuals without public or private insurance, were both less likely to experience hospitalization (2.0 percentage point, *P* = .01; 2.4 percentage point, *P* = .01) in the US. Analysis on the effect of insurance on hospitalization was not applicable for Taiwan sample as variation in insurance status was too small. Supplementary file 4 presented the results from additional analyses of the other outcomes associated with delayed care in the US from NHIS analytical sample.

## Discussion

 This study examined associations between chronic condition status and outcomes among individuals in the United States and Taiwan. Individuals with one or more chronic condition in the US were more likely to delay care, while their counterparts in Taiwan were less likely to do so. The relationship between chronic condition status and inpatient care resource use was generally similar in the US and Taiwan.

 The relationship between burden of chronic conditions and access to care is theoretically bidirectional. On one hand, individuals with MCCs need more medical care but are usually under limited financial resources; therefore, the “competing demand hypothesis” implies they may need to choose certain types of care over others, resulting in delays or even missing some necessary care at all.^17,29,30^ Additionally, in a healthcare system where care is fragmented, individuals with MCCs may delay needed care due to their time constraint, as they need to travel to different providers or to seek different types of care.^31,32^ These circumstances are consistent with evidence from the US, where individuals with MCCs can experience greater difficulty accessing medical services.^33^

 On the other hand, an inverse relationship between chronic conditions and delays in care could reflect environments in which care is less fragmented and/or more accessible. In such settings, “the surveillance hypothesis” suggests that patients with comorbid illnesses are screened more frequent or more likely to utilize care because they have more frequent contact with the medical care system.^17^ Accordingly, compared to those without any chronic conditions, individuals with MCCs may be more connected with the health system, more likely to be reminded for receiving needed care, and therefore less likely to delay their care. These circumstances are consistent with evidence from Taiwan in this study, where individuals with MCCs benefit from a longstanding single-payer system, universal national health insurance.

 Over the past three decades, the Taiwan national health insurance system has achieved its goals of ensuring access to care with an acceptable prices and quality of care.^34^ The system has significantly increased the convenience of seeking medical care. Compared to individuals with MCCs in other systems, perhaps this high-need population in Taiwan are less likely to hesitate to seek medical care, especially in a system where medical care is extremely convenient and affordable.^35^

 While the measures of delayed care differ between the two countries, the cross-national comparison remains methodologically justifiable and robust. First, the approach in this study represents a conservative comparison. The TSCS measure captures a broader scope of delayed care—encompassing both financial and non-financial reasons, whereas the NHIS measure is a subset restricted specifically to financial barriers. By comparing the broader Taiwan measure against the narrower US measure, we likely underestimate rather than overstate the US-Taiwan disparity. The fact that the effect of MCCs on cost-related delayed care in the US remains significantly higher than the effect on all-cause delayed care in Taiwan underscores the severity of financial barriers in the US system. Second, to address potential measurement bias, we incorporated three additional NHIS questions into our sensitivity analyses; the results remained consistent, further validating the robustness of our findings (See Supplementary file 4).

 The results indicate that MCC status is associated with a higher likelihood of hospitalization in both the US and Taiwan. In the US specifically, MCC status was associated with both delayed care and higher hospitalization. While this may appear paradoxical at the first glance, these findings are in fact consistent and reflect a broader challenge in MCC management. Delayed care and hospitalization represent different stages in the care continuum. Delayed care reflects barriers to accessing timely outpatient or primary care, and for individuals with MCC, such delays can lead to worsening symptoms or complications. As a result, the higher hospitalization rates observed in the US are likely a downstream consequence of delayed outpatient care rather than a contradiction. In contrast, the higher hospitalization rates observed among individuals with MCCs in Taiwan should be interpreted with caution and might be fundamentally different with in the US. Rather than signaling limited access or delayed care, these patterns may reflect features of Taiwan’s health system—such as the absence of a gatekeeping mechanism and the fragmented structure of primary care. Under Taiwan’s single-payer system, patients with MCCs can readily bypass primary care and seek hospital-based care directly. Thus, elevated hospitalization rates in Taiwan may indicate overreliance on secondary care or inefficiencies in care coordination, rather than the unmet needs–driven hospitalizations seen in the US.

 Lessons from Taiwan could be instructive for the US healthcare system, where national health insurance guaranteed all citizens with affordable and accessible care. Meanwhile, policy and practice leaders continue to use value-based payment and other national and regional insurance reforms, such as patient-centered pay-for-performance programs that incorporate diabetes and chronic kidney disease care, to promote more coordinated, less fragmented care. On the other hand, however, lessons from the US could be instructive for the Taiwan healthcare system, where leaders are grappling with budgetary pressures in the national insurance system and widespread of supplemental private health insurance, which nearly 71.1% of the population now possesses above and beyond public insurance.^36^

 The influence of health insurance profile on delays in care is significant in the US but less impactful in Taiwan. Our findings are consistent with prior work about the impact of insurance coverage on access to care in the US.^37-39^ While many factors may contribute to this divergence, a plausible explanation lies in the systemic differences between the healthcare frameworks of Taiwan and the US, encompassing national healthcare policies, insurance structures, and patient financial incentives. These variations likely exert a substantial influence on healthcare accessibility, thereby resulting in disparate associations between chronic condition status and delays in care. Given the ongoing expansion of private health insurance plans in Taiwan, it is imperative to observe whether diffusion of private insurance affects healthcare access, delays in care, or hospitalization, both in the short-term and long run.

 Lastly, it is worth noting that both surveys analyzed in this study were conducted in 2021, a year marked by the significant impact of COVID-19 in the US and began to affect Taiwan as well. The COVID-19 pandemic has had a significant impact on the healthcare system and the economy,^40^ leading to widespread illness, death, and disruption of daily life. During the pandemic, due to the rapid increase in the number of cases in the early stage of the epidemic, most medical care was focused on caring for COVID-19–patients, which makes medical supply to other diseases even scarcer. For individuals with MCCs who already face substantial barriers to accessing healthcare and have high medical needs, the impact of COVID-19 was particularly pronounced and cannot be overlooked.

###  Limitations

 This study had limitations. First, while many factors can lead to delayed medical care, data limitations preclude more in-depth analysis of specific reasons for delaying care. To account for this concern, we chose the other three questions in NHIS to test the sensitivity of this study. The findings could be found in Supplementary file 4. Second, study findings should be contextualized within limitations of self-reported survey data (eg, recall bias). However, our analysis follows precedent in using national surveys to evaluate healthcare choices and behaviors. For example, prior studies have used the NHIS to examine whether the use of complementary and alternative medicine is associated with health behaviors or risk factors known to influence health status^41^ and to analyze the health behaviors of specific populations.^42^ Third, the cross-sectional survey data limits the ability to make causal inferences. The relationship between MCCs and delayed care may be bidirectional; for instance, individuals who delay care may subsequently develop more comorbidities due to untreated conditions. Our findings also should be interpreted as a parallel comparison rather than a direct one. While we prioritized functional equivalence in most variables across the two surveys, unmeasured social and cultural factors may still influence survey responses. Consequently, the results should be interpreted with caution, and policy conclusions drawn from one setting may not be fully generalizable to the other without considering these contextual differences. Fourth, although our analysis included ten common chronic conditions, the findings may not generalize to other conditions. In addition, the NHIS and TSCS rely on different survey instruments and disease lists, which introduces challenges for direct comparison. To minimize this concern, we restricted our analyses to the ten chronic conditions that were consistently available in both datasets. Any remaining difference in condition lists would likely have only a small impact—primarily by misclassifying some individuals with multiple conditions as having fewer conditions. Such misclassification generally biases estimates toward the null. Next, data missing and lack of survey weighting parameters in the TSCS should be considered by interpreting study results. Also, data on some covariates, such as race and ethnicity, were not available in the Taiwan dataset in a format comparable to the US data. Finally, survey data were collected after the beginning of COVID-19 during a time of significant healthcare and social disruption. While data from 2021 do not reflect the most acute and disruptive phase of the pandemic, future work should assess dynamics in more recent, subsequent years.

## Conclusions

 This analysis used data from two distinct national healthcare systems to demonstrate different potential relationships between multimorbidity and delays in care. Ultimately, our study findings could imply that a bidirectional effect of MCC on likelihood of delaying needed care. Given ongoing changes in both regions – reforms that seek to reduce fragmentation and improve care coordination in the US; growing prevalence of, and risk for fragmented under, both public and private payers in Taiwan – findings from this study are potentially instructive beyond their national contexts for policy and healthcare system leaders.

## Acknowledgements

 Preliminary results of this study were presented at Academyhealth Annual Research Meeting 2024, Baltimore, Maryland, USA.

## Disclosure of artificial intelligence (AI) use

 Not applicable.

## Ethical issues

 Ethical approval was not necessary for this study as it relied exclusively on the analysis of publicly available, non-identified secondary data (National Health Interview Survey and TSCS).

## Conflicts of interest

 Authors declare that they have no conflicts of interest.

## Supplementary files


Supplementary file 1. Outcome Variables and Their Original Questions on TSCS/NHIS.

Supplementary file 2. Sample Selection for Multivariable Logistic Regression on TSCS/NHIS.

Supplementary file 3. The Frequency of Top Three Combinations of Diseases in Individuals With Two Chronic Conditions in the Sample of Two Chronic Conditions.

Supplementary file 4. Additional Analysis for the US Sample - Weighted Marginal Effect Estimates of MCC on Delayed Medical Care Due to COVID-19 and Did Not Get Care Due to Cost or COVID-19.
