# Supplementary material for: Multiple Chronic Conditions, Delayed Medical Care and Hospitalization: A Comparison Between the United States and Taiwan
Source: Int J Health Policy Manag. 2026 Feb 17;15:9164. doi: 10.34172/ijhpm.9164 (PMC13034188; doi:10.34172/ijhpm.9164)
Supplement: Supplementary file 1 — Outcome Variables and Their Original Questions on TSCS/NHIS. [file ijhpm-15-9164-s001.pdf]

Article title: Multiple Chronic Conditions, Delayed Medical Care and Hospitalization: A Comparison Between the United States and Taiwan

Journal name: International Journal of Health Policy and Management (IJHPM)

Authors' information: Chen-Yang Wang[1], Ching-Ching Claire Lin[1,2,3]*, Raymond N. Kuo[1,2], Joshua M. Liao[4]

[1]Institute of Health Policy and Management, College of Public Health, National Taiwan University, Taipei, Taiwan.

[2]Population Health Research Center, National Taiwan University, Taipei, Taiwan.

[3]Master of Public Health Degree Program, College of Public Health, National Taiwan University, Taipei, Taiwan.

[4]Department of Internal Medicine, UT Southwestern Medical Center, Dallas, TX, USA.

***Correspondence to:** Ching-Ching Claire Lin; Email: ccclin@ntu.edu.tw

Citation: Wang CY, Lin CCC, Kuo RN, Liao JM. Multiple chronic conditions, delayed medical care and hospitalization: a comparison between the United States and Taiwan. Int J Health Policy Manag. 2026;15:9164. doi:10.34172/ijhpm.9164

Supplementary file 1. Outcome Variables and Their Original Questions on TSCS/NHIS

variable	TSCS questions (translated from Mandarin)	Possible responses for TSCS (translated from Mandarin)	NHIS questions	Possible responses for NHIS
Delayed care	In the past 12 months, have you refrained from going to see a doctor?	1. Yes<br>2. No<br>3. Not ill or injured during the last 12m<br>4. Don't know	During the past 12 months, have you delayed getting medical care because of the cost?	1. yes<br>2. No<br>3. Refused<br>4. Not ascertained<br>5. Don’t know
			Was there any time when you delayed getting medical care because of the coronavirus pandemic?	1. yes<br>2. No<br>3. Refused<br>4. Not ascertained<br>5. Don’t know
			During the past 12 months, was there any time when you needed medical care, but did not get it because of the cost?	1. yes<br>2. No<br>3. Refused<br>4. Not ascertained<br>5. Don’t know
			Was there any time when you needed medical care for something other than coronavirus, but did not get it because of the	1. yes<br>2. No<br>3. Refused<br>4. Not ascertained

			coronavirus pandemic?	5. Don’t know
Inpatient use	Have you been in hospital or a clinic as an inpatient overnight?	1. Yes<br>2. No<br>3. Don’t know	During the past 12 months, have you been hospitalized overnight?	1. Yes<br>2. No<br>3. Refused<br>4. Not ascertained<br>5. Don’t know
