# Supplementary material for: Multiple Chronic Conditions, Delayed Medical Care and Hospitalization: A Comparison Between the United States and Taiwan
Source: Int J Health Policy Manag. 2026 Feb 17;15:9164. doi: 10.34172/ijhpm.9164 (PMC13034188; doi:10.34172/ijhpm.9164)
Supplement: Supplementary file 2 — Sample Selection for Multivariable Logistic Regression on TSCS/NHIS. [file ijhpm-15-9164-s002.pdf]

Article title: Multiple Chronic Conditions, Delayed Medical Care and Hospitalization: A Comparison Between the United States and Taiwan

Journal name: International Journal of Health Policy and Management (IJHPM)

Authors' information: Chen-Yang Wang[1], Ching-Ching Claire Lin[1,2,3]*, Raymond N. Kuo[1,2], Joshua M. Liao[4]

[1]Institute of Health Policy and Management, College of Public Health, National Taiwan University, Taipei, Taiwan.

[2]Population Health Research Center, National Taiwan University, Taipei, Taiwan.

[3]Master of Public Health Degree Program, College of Public Health, National Taiwan University, Taipei, Taiwan.

[4]Department of Internal Medicine, UT Southwestern Medical Center, Dallas, TX, USA.

***Correspondence to:** Ching-Ching Claire Lin; Email: ccclin@ntu.edu.tw

Citation: Wang CY, Lin CCC, Kuo RN, Liao JM. Multiple chronic conditions, delayed medical care and hospitalization: a comparison between the United States and Taiwan. Int J Health Policy Manag. 2026;15:9164.doi:10.34172/ijhpm.9164

Supplementary file 2. Sample Selection for Multivariable Logistic Regression on TSCS/NHIS

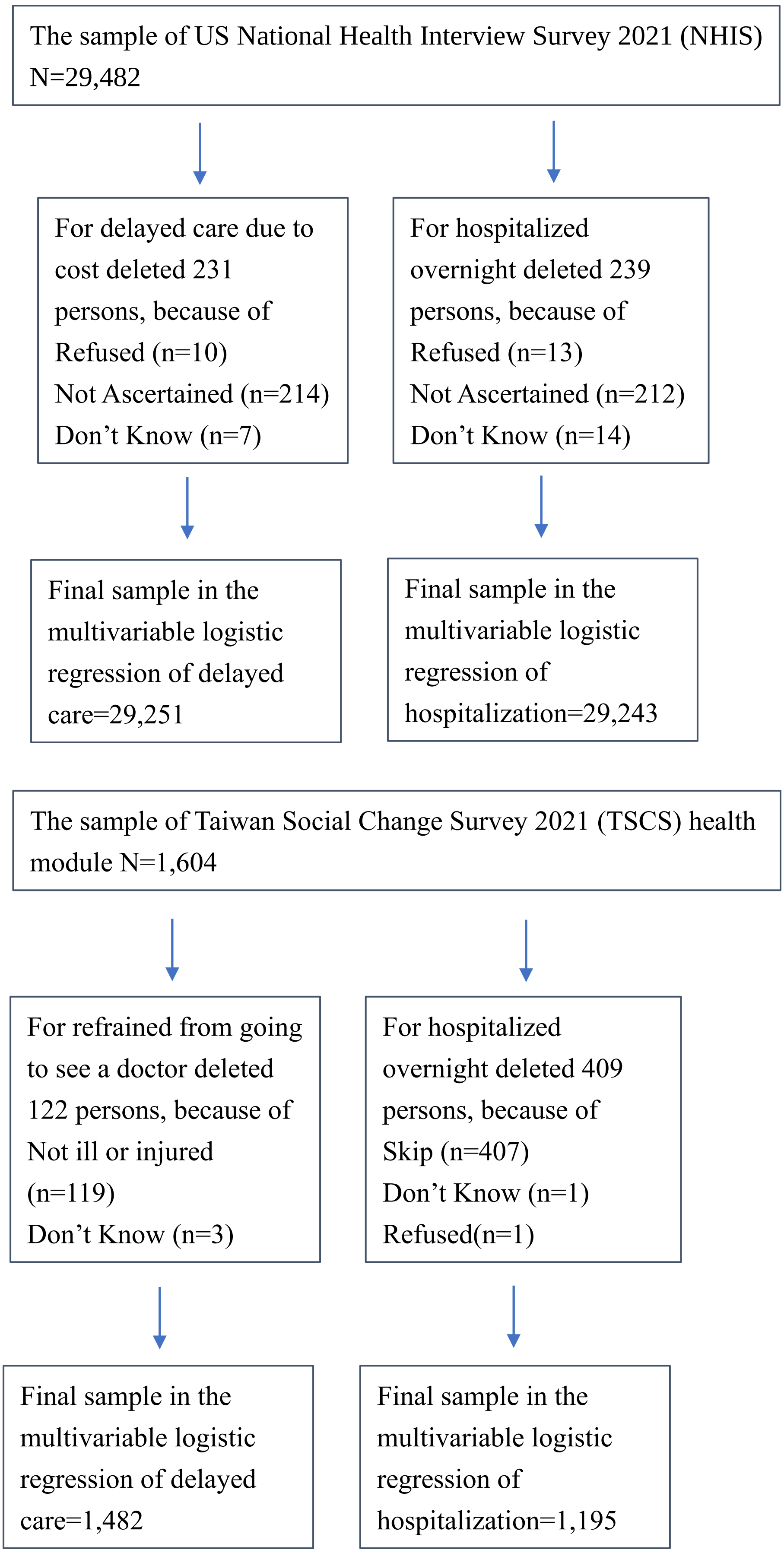
The sample of US National Health Interview Survey 2021 (NHIS) N=29,482
For delayed care due to cost deleted 231 persons, because of Refused (n=10) Not Ascertained (n=214) Don’t Know (n=7)
For hospitalized overnight deleted 239 persons, because of Refused (n=13) Not Ascertained (n=212) Don’t Know (n=14)
Final sample in the multivariable logistic regression of delayed care=29,251
Final sample in the multivariable logistic regression of hospitalization=29,243
The sample of Taiwan Social Change Survey 2021 (TSCS) health module N=1,604
For refrained from going to see a doctor deleted 122 persons, because of Not ill or injured (n=119) Don’t Know (n=3)
For hospitalized overnight deleted 409 persons, because of Skip (n=407) Don’t Know (n=1) Refused(n=1)
Final sample in the multivariable logistic regression of delayed care=1,482
Final sample in the multivariable logistic regression of hospitalization=1,195
