# Supplementary material for: Multiple Chronic Conditions, Delayed Medical Care and Hospitalization: A Comparison Between the United States and Taiwan
Source: Int J Health Policy Manag. 2026 Feb 17;15:9164. doi: 10.34172/ijhpm.9164 (PMC13034188; doi:10.34172/ijhpm.9164)
Supplement: Supplementary file 3 — The Frequency of Top Three Combinations of Diseases in Individuals With Two Chronic Conditions in the Sample of Two Chronic Conditions. [file ijhpm-15-9164-s003.pdf]

Article title: Multiple Chronic Conditions, Delayed Medical Care and Hospitalization: A Comparison Between the United States and Taiwan

Journal name: International Journal of Health Policy and Management (IJHPM)

Authors' information: Chen-Yang Wang[1], Ching-Ching Claire Lin[1,2,3]*, Raymond N. Kuo[1,2], Joshua M. Liao[4]

[1]Institute of Health Policy and Management, College of Public Health, National Taiwan University, Taipei, Taiwan.

[2]Population Health Research Center, National Taiwan University, Taipei, Taiwan.

[3]Master of Public Health Degree Program, College of Public Health, National Taiwan University, Taipei, Taiwan.

[4]Department of Internal Medicine, UT Southwestern Medical Center, Dallas, TX, USA.

***Correspondence to:** Ching-Ching Claire Lin; Email: ccclin@ntu.edu.tw

Citation: Wang CY, Lin CCC, Kuo RN, Liao JM. Multiple chronic conditions, delayed medical care and hospitalization: a comparison between the United States and Taiwan. Int J Health Policy Manag. 2026;15:9164.doi:10.34172/ijhpm.9164

Supplementary file 3. The Frequency of Top Three Combinations of Diseases in Individuals With Two Chronic Conditions in the Sample of Two Chronic Conditions

	Sample N	Weighted %		Sample N	Weighted %
Total	4,725	100%	Total	142	-
Hypertension & Arthritis	1,323	26.2%	Hypertension & Diabetes	41	28.2%
Hypertension & Diabetes	537	12.3%	Hypertension & Heart Disease	16	11.1%
Hypertension & Cancer	486	9.1%	Hypertension & Arthritis	6	4.2%
* The sample size of those with two chronic conditions in the US is 4,725; the sample size of those with two chronic conditions in Taiwan is 142.					
