# Supplementary material for: Multiple Chronic Conditions, Delayed Medical Care and Hospitalization: A Comparison Between the United States and Taiwan
Source: Int J Health Policy Manag. 2026 Feb 17;15:9164. doi: 10.34172/ijhpm.9164 (PMC13034188; doi:10.34172/ijhpm.9164)
Supplement: Supplementary file 4 — Additional Analysis for the US Sample - Weighted Marginal Effect Estimates of MCC on Delayed Medical Care Due to COVID-19 and Did Not Get Care Due to Cost or COVID-19. [file ijhpm-15-9164-s004.pdf]

Article title: Multiple Chronic Conditions, Delayed Medical Care and Hospitalization: A Comparison Between the United States and Taiwan

Journal name: International Journal of Health Policy and Management (IJHPM)

Authors' information: Chen-Yang Wang[1], Ching-Ching Claire Lin[1,2,3]*, Raymond N. Kuo[1,2], Joshua M. Liao[4]

[1]Institute of Health Policy and Management, College of Public Health, National Taiwan University, Taipei, Taiwan.

[2]Population Health Research Center, National Taiwan University, Taipei, Taiwan.

[3]Master of Public Health Degree Program, College of Public Health, National Taiwan University, Taipei, Taiwan.

[4]Department of Internal Medicine, UT Southwestern Medical Center, Dallas, TX, USA.

***Correspondence to:** Ching-Ching Claire Lin; Email: ccclin@ntu.edu.tw

Citation: Wang CY, Lin CCC, Kuo RN, Liao JM. Multiple chronic conditions, delayed medical care and hospitalization: a comparison between the United States and Taiwan. Int J Health Policy Manag. 2026;15:9164.doi:10.34172/ijhpm.9164

Supplementary file 4. Additional Analysis for the US Sample - Weighted Marginal Effect Estimates of MCC on Delayed Medical Care Due to COVID-19 and Did Not Get Care Due to Cost or COVID-19

Outcome Variables	United States<br>Delayed care due to Covid-19 in the past 12m (n=29,241)			Did not get care due to cost in the past 12m (n=29,234)			Did not get care due to Covid-19 in the past 12m (n=29,229)		
	**ME**	**SE**	**P-value**	**ME**	**SE**	**P-value**	**ME**	**SE**	**P-value**
**MCC status**									
No CC		[reference]			[reference]			[reference]	
With only one CC	0.059	0.007	<.001	0.015	0.004	<.001	0.039	0.006	<.001
MCC	0.085	0.008	<.001	0.039	0.005	<.001	0.066	0.007	<.001
**Age (65+)**	-0.061	0.009	<.001	-0.046	0.004	<.001	-0.060	0.007	<.001
**Male**	-0.073	0.005	<.001	-0.019	0.003	.04	-0.046	0.005	<.001
**General health status**									
Excellent		[reference]			[reference]			[reference]	
Very Good	0.041	0.006	<.001	0.011	0.004	.007	0.026	0.005	<.001
Good	0.062	0.007	<.001	0.030	0.005	<.001	0.044	0.006	<.001
Fair/Poor	0.121	0.011	<.001	0.077	0.008	<.001	0.100	0.009	<.001
**Urban**	0.024	0.007	.001	-0.003	0.005	.53	0.008	0.007	.23
**Region**									
Northeast		[reference]			[reference]			[reference]	
Midwest	-0.038	0.010	<.001	0.007	0.006	.26	-0.027	0.007	<.001

South	-0.036	0.009	<.001	0.010	0.006	.10	-0.018	0.007	.01
West	0.007	0.010	.49	0.012	0.006	.07	0.012	0.008	.11
**Education**									
Under 12 grades		[reference]			[reference]			[reference]	
GED/High school	0.008	0.009	.38	0.003	0.009	.65	0.013	0.007	.056
College	0.039	0.010	<.001	0.012	0.006	.057	0.043	0.008	<.001
Bachelor's degree	0.080	0.010	<.001	0.015	0.006	.01	0.060	0.007	<.001
Graduate	0.135	0.012	<.001	0.004	0.007	.63	0.097	0.009	<.001
**Race and ethnicity**									
Non-Hispanic White		[reference]			[reference]			[reference]	
Hispanic	-0.001	0.009	.93	-0.011	0.005	.02	0.002	0.007	.75
Non-Hispanic Black	-0.029	0.009	.002	-0.007	0.005	.21	-0.015	0.007	.04
Others	-0.010	0.010	.33	-0.017	0.006	.004	-0.009	0.008	.23
**Marital status**	0.009	0.006	.13	-0.001	0.003	.78	0.008	0.004	.07
**Employment status**	-0.017	0.007	.02	0.004	0.004	.33	-0.018	0.006	.001
**Insurance type**									
Public only		[reference]			[reference]			[reference]	
Private only	0.003	0.013	.80	0.010	0.008	.19	-0.011	0.010	.28
Public + Private	-0.011	0.010	.24	-0.018	0.006	.003	-0.013	0.008	.09
Non-public Non-	-0.014	0.015	.34	0.081	0.010	<.001	-0.006	0.012	.63

private									
**Household income**									
<150%		[reference]			[reference]			[reference]	
150% to <250%	-0.016	0.010	.10	-0.009	0.007	.18	-0.018	0.009	.03
250% to <400%	-0.017	0.009	.08	-0.031	0.007	<.001	-0.024	0.008	.006
400% or more	0.011	0.010	.27	-0.052	0.006	<.001	-0.013	0.008	.13
**Have a usual place to go for care**									
No		[reference]			[reference]			[reference]	
Yes	0.022	0.010	.03	-0.045	0.007	<.001	0.013	0.008	.11
More than 1	0.076	0.024	.001	-0.016	0.016	.32	0.084	0.023	<.001
